# The effectiveness of an educational intervention for sodium restriction in patients with hypertension: study protocol for a randomized controlled trial

**DOI:** 10.1186/s13063-017-2091-4

**Published:** 2017-07-21

**Authors:** Marcela Perdomo Rodrigues, Luciana Kaercher John dos Santos, Flavio Danni Fuchs, Sandra Costa Fuchs, Leila Beltrami Moreira

**Affiliations:** 1 Postgraduate Studies Program in Cardiology, Universidade Federal do Rio Grande do Sul, Porto Alegre, RS Brazil; 2 Division of Cardiology, Hospital de Clínicas de Porto Alegre, Porto Alegre, Brazil

**Keywords:** Hypertension, Sodium restriction, Educational intervention, Dietary sodium restriction questionnaire (DSRQ), Low-sodium diet, Adherence

## Abstract

**Background:**

The effectiveness of nonpharmacological interventions in blood pressure reduction has been evidenced by several studies. Nevertheless, as adherence to a low-sodium diet is poor, interventions regarding habit changing should be of a motivational nature in order to develop the ability of overcoming obstacles regarding sodium-restriction behavior.

The present study aims to describe the protocol and randomization of a clinical trial design in order to evaluate the effectiveness of an educational intervention based on Dietary Sodium Restriction Questionnaire (DSRQ) scores. The effectiveness measures are the DSRQ score variation and reduction in urinary sodium values from baseline to after 2 and 6 months.

**Methods/design:**

This parallel, randomized clinical trial will include 120 participants, recruited and randomized as follows: 60 of them to be allocated to a sodium-restriction educational intervention group whose results are based on the DSRQ application; and the other 60 allocated to a control group with usual care. Educational orientation and usual care sessions will be conducted once a month for a period of 6 months. Both spot urine collection – estimating sodium intake – and the DSRQ will be applied at the baseline, in the eighth week and at the end of the follow-up. There will also be blood collection and 24-h ambulatory blood pressure monitoring (ABPM) at the beginning and end of the follow-up. Anthropometric measurements, blood pressure measurement and 24-h food recall will be collected during follow-up.

**Discussion:**

The study “The effectiveness of an educational intervention to sodium restriction in patients with hypertension” is based on the results of the DSRQ application, whose objective is to evaluate aspects related to nonadherence to the recommendation of a low-sodium diet, identifying adherence barriers and facilitators, contributing to the planning of interventions for improving the adoption of a low-sodium diet and, consequently, hypertension control.

**Trial registration number:**

ClinicalTrials.gov, Identifier: NCT02848690. Registered retrospectively on 27 July 2016.

**Electronic supplementary material:**

The online version of this article (doi:10.1186/s13063-017-2091-4) contains supplementary material, which is available to authorized users.

## Background

Clinic trials have demonstrated the efficacy of nutritional interventions in the reduction of blood pressure [1–6]. According to a meta-analysis, sodium-intake restriction has shown effectiveness in reducing blood pressure in hypertensive and normotensive individuals [7]. Even salt-restriction diets, such as DASH (Dietary Approaches to Stop Hypertension) [1, 2] and the Mediterranean diet [6], were associated with a decrease in blood pressure levels. The guidelines to reduce salt intake to 5–6 g/day should, therefore, have a major effect on blood pressure, but a further reduction to 3 g/day will have an even greater effect and should become the long-term target for population salt intake [7].

In contrast, evidence has demonstrated that there is an increased risk of cardiovascular disease events and deaths associated with 24-h urinary sodium excretion of less than 3 g/day in both hypertensive and normotensive individuals. However, high-sodium intake (greater than 6 g/day) was associated with an increased risk in individuals with hypertension. These data suggest that lowering sodium intake is best targeted at populations with hypertension who consume high-sodium diets [8]. Thus, current evidence suggests a recommendation for moderate sodium intake in the general population (3–5 g/day), targeting the lower end of the moderate range among those with hypertension [9].

According to hypertension treatment guidelines, a daily intake of approximately 3.7 g/day (1500 mg of sodium) is recommended [10, 11]. Furthermore, a reduction of 2.0–4.6 g/day of salt can result in clinically significant lower systolic blood pressure [7]. However, low-sodium diet adherence is poor, since most patients are asymptomatic and present difficulties in adopting and perceiving the benefits of reduced sodium intake, usually considering them too restrictive [12]. Also, there is no measure considered as “gold standard” for assessing treatment adherence, making the perception of nonadherence difficult for health professionals [13].

Interventions for behavior change should develop motivation to achieve goals such as reducing salt addition during meal preparation as well as the consumption of salty food; besides that, health professionals should consider the need to incorporate strategies to improve the necessary skills to overcome obstacles related to salt-intake-restriction behavior; thus, help transforming intention into action and strengthen such intentions by improving the perception of self-efficacy and habit change [14].

Only providing information about food to be avoided would not be enough to change this behavior, it is also necessary to help patients find out other kinds of food, replacing salty aliments with a healthier diet but with the same level of dietary pleasure [15]. Thus, a hedonic change to lower salt levels may be achieved by gradual exposure to food with low-salt content, resulting in a reduction of the salty taste threshold [16]. Furthermore, simple advising, though in clinical environments and administered in each visit, tends to show low efficiency; moreover, even in intensive counseling, only 20 to 40% of the participants in clinical trials of sodium restriction reduced their consumption to below the maximum recommended limit of 2300 mg/day (100 mmol/day) [3, 17, 18].

The Dietary Sodium Restriction Questionnaire (DSRQ) was developed due to the lack of instruments for measuring low-sodium diets among patients with heart failure (HF), in order to improve adherence to a low-sodium diet [19]. This questionnaire aims to help identify facilitators and barriers to adherence to a low-sodium diet, which is useful in clinical practice in order to guide the development of interventions for patient education and counseling [19].

Adherence to a lifestyle modification program, in order to modify health behavior, is very difficult because there are many barriers to behavioral change, such as knowledge about the disease, health beliefs, relationships with health professionals, as well as a variety of social and environmental factors, including financial difficulties, lack of transportation, and long periods of waiting in hospitals and clinics [20].

### Objective

This study aims to describe the protocol and randomization of the clinical trial design in order to evaluate the effectiveness of an educational intervention based on DSRQ scores. The effectiveness measures are DSRQ score variation and urinary reduction in sodium values from baseline and after 2 and 6 months.

### Hypothesis

Patients with hypertension who participate in the intervention group show low-sodium diet adherence, which decreases blood pressure values assessed by ABPM and sodium urine excretion values after 2 and 6 months in comparison to the control group.

## Methods/design

### Study design

**Fig. 1:**
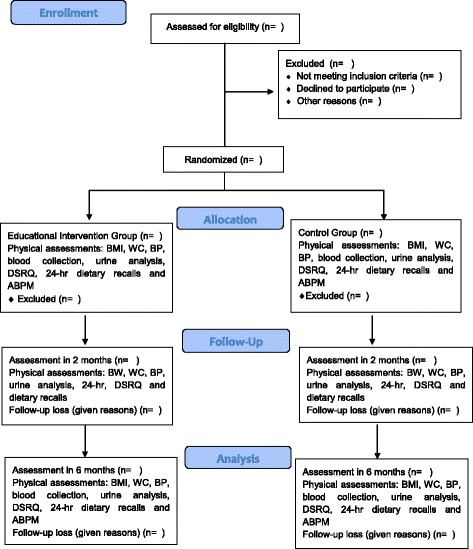
General overview of study design

**Fig. 2:**
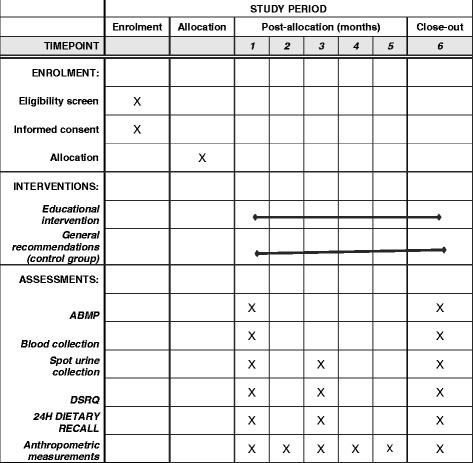
Study timeline and schedule of enrollment, interventions and assessments

This study is randomized, single-center, parallel clinical trial, with a follow-up period of 6 months. The hypertensive outpatients of Hospital de Clínicas de Porto Alegre (HCPA), will be recruited and then invited by telephone to participate in a study. After a baseline evaluation, the participants will be randomly allocated into two groups: (1) an educational intervention group and (2) a control group; and monitored monthly. The flow of the study design is presented in Fig. 1. The study timeline and schedule of enrollment, interventions and assessments (Standard Protocol Items: Recommendations for Interventional Trials (SPIRIT) figure) and the SPIRIT Checklist are provided as Fig. 2 and Additional file 1, respectively.

### Eligibility criteria

The study will include female and male individuals, aged from 40 to 80 years, who are in treatment and being monitored at the hypertensive outpatient department of HCPA. Participants must not have been monitored by a nutritionist or followed a nutritional orientation for more than 6 months.

Participants will be excluded from the study if they fit into one or more of the following criteria*:* pregnancy or lactation; gastrointestinal tract disease; inflammatory disease; chemotherapy treatment; diabetes diagnosis; incapacity to engage in an interview and/or to participate in the intervention program without the need of third-party involvement.

### Randomization, allocation and confidentiality

The first visit will consist of: (1) eligibility confirmation, (2) informed consent signature and (3) sociodemographic and clinic data collection. After this initial visit, patients will be allocated to a control group or an intervention group, according to a randomized code. An independent person not involved in this study will possess a list with the computer-generated randomization codes. Randomization sequence will be generated in blocks of 6, according to specific software, as further explained. Participants will be allocated to one of the two groups, using numbered envelopes, by simple random sampling determined by a randomization list.

### Control group

Participants allocated to the control group will attend a nutritionist consultation regarding general recommendations for hypertension, such as increasing their consumption of fruits and vegetables, reducing salt intake, avoiding processed food and high-sodium food, reducing body weight if Body Mass Index (BMI) >25 Kg.m^2^ and reducing their consumption of alcoholic beverages, and will be provided with an explanatory folder about hypertension. During 6 months, participants of the control group will have their blood pressure (BP), body weight and waist circumference measures assessed monthly. During the same period of time, participants will be also asked if they have any questions about food.

### Educational intervention group

The intervention sessions are designed according to the subscales of the DSRQ [21], and they will all be developed and performed by the nutritionist, who is the investigator responsible for the educational intervention, based on the Theory of Planned Behavior (TPB) [22].

Participants allocated to the educational intervention group will attend a dietitian consultation and receive dietary planning based on a diet rich in fruits and vegetables, low in fat, low in processed food and high in nonfat dairy products. During 6 months they will have monthly education sessions on sodium restriction to enhance and follow the dietary planning.

Educational intervention about sodium restriction will be based on the results of the DSRQ application. The instrument will be applied at the baseline to guide intervention activities and strategies and this will be applied again after 2 and 6 months and at the end of the follow-up to evaluate low-sodium dietary adherence.

The interventional sessions will be face-to-face, 1-h-long and with the aim to encourage and motivate low-sodium diet adherence, using approaches that provide individual skills to achieve the goals (sodium restriction), developing changes in behavior and monitoring the progress towards the skills needed to reduce the barriers and difficulties of sodium-restricted dietary adherence. Table 1 describes the instructions for the intervention in each visit.

**Table 1:** Educational intervention review

Intervention	Educational intervention group	Control group
Baseline	Informed consent signature and collection of demographic and anthropometric data, blood pressure measurement, DSRQ application, antihypertensive medications used and randomization. Spot urine collection and blood collection.	
	Orientation about low-sodium diet and individualized food plan	General orientation on hypertension and sodium restriction
2nd month	Hypertension: concept, intervention aims, low-sodium diet importance.	
3rd month	Choosing low-sodium food in restaurants and supermarkets (dependent behavior) Instructions for completing a 3-day food record. DSRQ application and spot urine collection.	DSRQ application and spot urine collection
4th month	Intervention based on the result of the food record (attitude) Third-party influence on sodium restriction: family support importance (subjective norm)	
5th month	Preparation of low-sodium food: use of natural spices, salt substitutes (perceived behavioral control) End of intervention: review of items, motivation to sodium-restriction maintenance	
6th month	The end of follow-up evaluation: anthropometric assessment, blood pressure measurement and DSRQ application. Spot urine collection and blood collection	

*DSRQ* Dietary Sodium Restriction Questionnaire

The activities will be developed according to the instrumental subscales [23]:

1. Attitude – explanation to understand the low-sodium dietary importance
2. Subjective norm – sessions about the influence of family and others in choices and food preparation. Family support in sodium-restriction-diet adherence
3. Perceived control – learning sessions to increase information about food choices, cooking or preparing food without salt, evaluating recipes and making suggestions for changes to low-sodium food, learning to read labels (strategies to increase low-sodium diet adherence)
4. Dependent behavior – learning sessions on the amount of sodium in food, including salt quantity demonstrations, low-sodium food selection, changes in food choices in restaurants, being aware of the need to change both taste and food preferences

### Study variables and methods of assessment

The research team will be responsible for the triage and selection of eligible participants, and three members of the team will obtain the written informed consent signatures prior to entering the study. Researchers will not be blinded to participant intervention. The investigators will be trained to perform anthropometric standardization and BP measurements as well as the application of questionnaires. A laboratory technician will collect both spot urine and blood collection after 12-h fasting and the samples will be forwarded for analysis.

#### Blood pressure (BP)

BP will be measured with the participant seated quietly, with feet on the floor and arm supported at heart level, according to guidelines [24]. The cuff size will be used according to arm circumference; two measurements will be taken and the average recorded. BP will be measured at all visits.

#### Anthropometric measurements

The anthropometric measurements to be collected will be body weight (BW), height, waist circumference (WC) (midpoint between the last rib and the superior edge of the iliac crest) and Body Mass Index (BMI) in all visits.

#### Blood collection

Blood collection will be analyzed after 12-h fasting at baseline and at the end of the follow-up for clinical assessment (total cholesterol, high-density lipoprotein (HDL)-cholesterol, low-density lipoprotein (LDL)-cholesterol, triglycerides, glucose, sodium and potassium).

#### Spot urine analysis

Spot urine will be collected to estimate sodium intake, at baseline, after 2 months and at the end of the follow-up (6 months).

#### 24-h ambulatory blood pressure monitoring (ABPM)

AMPB will be performed using Spacelabs 90207 devices (Redmond, WA, USA). The protocol includes BP measurements every 15 min during daytime (6:00 a.m. to 10:00 p.m.) and every 20 min during nighttime (10:00 p.m. to 6:00 a.m.). ABPM will be considered satisfactory if at least 16 valid readings during daytime and 8 valid readings during nighttime are obtained [25]. Participants will be evaluated by ABPM at the baseline and at the end of the follow-up (6 months) in order to register BP during both sleep and wakefulness.

#### Dietary Sodium Restriction Questionnaire (DSRQ)

The participants will answer the DSRQ for the assessment of sodium-restriction adherence at the baseline, 2 months and at the end of the follow-up. This application will guide the educational intervention.

#### Dietetic assessments

The participants will answer the 24-h dietary recall for monitoring low-sodium diet adherence, detailing of food and portion consumed, as well as the ingredients, including information on time, place and quantity.

### Study outcomes

Primary outcomes: changes from baseline, and after 2 and 6 months, in DSRQ score, urinary sodium values and mean BP as assessed by ABPM.

Secondary outcomes: questionnaire sensitivity and specificity by the comparison of DSRQ scores to urinary sodium values.

### Sample size

Sodium reduction is assessed by urinary sodium values and estimated sodium intake; thus, sample size was calculated according to sodium reduction. The DSRQ was developed in order to improve sodium-restriction adherence [19], the more adherence the less sodium intake, which, in turn, reduces urinary sodium values and mean BP [26]. Sample size was estimated based on the data of the previous study with the same hypertensive population, with an estimated mean of 3900.98 ± 1602.2 mg/day (168.5 mmol) of sodium [27]. We expect to obtain a 100-mmol (2300-mg/day) reduction with the intervention; moreover, in order to detect a 40-mmol urinary sodium difference, a standard deviation of approximately 69.2 mmol, an alpha error of 5% and power of 80%, the study will include 48 participants in each group.

We opted for a 20% increase over the initial number to account for follow-up loss or subject withdrawal so that we arrived at a total of 120 participants included and assigned to either the intervention or the control group.

### Statistical analysis plan

The sample characteristics will be presented by descriptive statistics, and the results will be expressed as mean, standard deviation and percentage. Pearson’s chi-square test and Student’s *t* test will be used to compare categorical and continuous variables, respectively, between groups. In order to analyze educational intervention effectiveness, deltas of sodium urinary excretion will be compared by analysis of covariance.

The questionnaire sensitivity and specificity to identify low-sodium-restriction adherence will be evaluated comparing DSRQ score results to sodium values estimated by urine collection and ROC curve.

All data will be analyzed according to the intention-to-treat principle. The statistics analysis will be carried out in PASW Statistics 18® (International Business Machines Corp., Armonk, NY, USA). A *P* value < 0.05 will be considered statistically significant.

## Discussion

This trial evaluates the hypothesis that patients with hypertension, when participating in an educational intervention for sodium restriction, improve their low-sodium diet adherence, thus decreasing blood pressure values and sodium urinary excretion. The educational intervention for sodium restriction will be planned based on the application of the DSRQ in patients with hypertension. The DSRQ was adapted to Brazilian Portuguese [28] and validated for heart failure (HF) [29] to help identifying facilitators and barriers to sodium-restriction adherence, and to be useful in clinical practice to guide the development of educational and counseling interventions to patients [19]. Considering the importance of assessing salt-restriction adherence and the lack of instruments for easy application in the care routine, the DSRQ was validated for hypertension [21] with the purpose of being an instrument to assess low-sodium diet adherence which can contribute to the planning of educational interventions in patients with hypertension.

The DSRQ was developed for patients with HF [19] and applied to evaluate the effectiveness of an educational intervention, based on the TPB. It has been demonstrated that such an intervention was effective to reduce sodium intake compared to the control group after 6 months. It was also found out that the attitude towards a low-sodium diet improved during the 6 weeks of the intervention. Concluding, educational programs that are carefully designed have the potential to produce the desired results, such as low-sodium diet adherence in patients with HF [26].

For any health recommendation to be successful it is necessary to overcome behavioral barriers to dietary change and the influence of interindividual variation in dietary response [30]. However, it is not yet clear what theoretical basis is the best to select intervention methods and strategies; nevertheless, TPB is the theory most used to understand and predict health behaviors, including food-related behaviors [22]. According to TPB, behavior is a function of intention to act and the perception of control over behavior, the intention being determined by three variables: attitudes related to behavior, subjective norm and perceived behavioral control [22].

People with higher perception of their capabilities usually perceive the difficulties as problems to be experienced and not as threats to be avoided; on the other hand, people who are hesitant regarding their own capacities, perceive difficult tasks as threatening, and thus, the more confident that the person is in their ability to follow a healthy and low-sodium diet, the more likely they are to follow this recommendation [14].

### Potential biases

This study has some limitations. It is difficult to target a specific behavior concerning salt intake because salt can originate from several natural sources or be added to food during cooking or food processing. The instrument is self-reported, and self-reported behavior measurements can present a range of bias such as data credibility. The open-label design can introduce some potential for biases and the sample size limits the external validity.

### Trial status

Participant recruitment of this randomized clinical trial begun in November 2015.

## Additional file

Additional file 1: SPIRIT 2013 Checklist: recommended items to address in a clinical trial protocol and related documents*. (DOC 120 kb)

## Abbreviations

ABPM: 24-h ambulatory blood pressure monitoring
BMI: Body Mass Index
BP: Blood pressure
DSRQ: Dietary Sodium Restriction Questionnaire
HF: Heart failure
TPB: Theory of Planned Behavior
WC: Waist circumference

## References

[1] Appel LJ, Moore TJ, Obarzanek E, Vollmer WM, Svetkey LP, Sacks FM (1997). A clinical trial of the effects of dietary patterns on blood pressure. DASH Collaborative Research Group. N Engl J Med.

[2] Sacks FM, Svetkey LP, Vollmer WM, Appel LJ, Bray GA, Harsha D, DASH-Sodium Collaborative Research Group (2001). Effects on blood pressure of reduced dietary sodium and the Dietary Approaches to Stop Hypertension (DASH) diet. DASH-Sodium Collaborative Research Group. N Engl J Med.

[3] Appel LJ, Champagne CM, Harsha DW, Cooper LS, Obarzanek E, Elmer PJ, Stevens VJ (2003). Effects of comprehensive lifestyle modification on blood pressure control: main results of the PREMIER clinical trial. JAMA..

[4] Hinderliter A, Sherwood A, Craighead L, Lin P, Watkins LL, Babyak MA, Blumenthal JA (2014). The long term effects of lifestyle chance on blood pressure: one year follow-up of the ENCORE Study. Am J Hypertens..

[5] Cook NR, Cutler JA, Obarzanek E, Buring JE, Rexrode KM, Kumanyka SK, Appel LJ, Whelton PK (2007). Long term effects of dietary sodium reduction on cardiovascular disease outcomes: observational follow-up of the trials of hypertension prevention (TOHP). BMJ..

[6] Toledo E, Hu FB, Estruch R, Buil-Cosiales P, Corella D, Salas-Salvadó J (2013). Effect of the Mediterranean diet on blood pressure in the PREDIMED trial: results from a randomized controlled trial. BMC..

[7] He FJ, MacGregor GA. Effect of longer-term modest salt reduction on blood pressure. Cochrane Database Syst Rev. 2004;(3):CD004937. 10.1002/14651858.CD004937 15266549

[8] Mente A, O’Donnell M, Rangarajan S, Dagenais G, Lear S, McQueen M (2016). Associations of urinary sodium excretion with cardiovascular events in individuals with and without hypertension: a pooled analysis of data from four studies. Lancet..

[9] O’Donnell M, Mente A, Yusuf S (2015). Sodium intake and cardiovascular health. Circ Res.

[10] Lichtenstein AH, Appel LJ, Brands M, Carnethon M, Daniels S, Franch HA (2006). Diet and lifestyle recommendations revision 2006: a scientific statement from the American Heart Association Nutrition Committee. Circulation..

[11] Appel LJ, Frohlich ED, Hall JE, Pearson TA, Sacco RL, Seals DR (2011). The importance of population-wide sodium reduction as a means to prevent cardiovascular disease and stroke: a call to action from the American Heart Association. Circulation..

[12] Evers SE, Bass M, Donner A, McWhinney IR (1987). Lack of impact of salt restriction advice on hypertensive patients. Prev Med..

[13] World Health Organization. Adherence to long-term therapies: evidence for action. Geneva: WHO Library Cataloguing-in-Publication Data; 2003.

[14] Cornélio ME, Gallani MCBJ, Godin G, Rodrigues RCM, NadruzJr W, Mendez RDR (2012). Behavioral determinants of salt consumption among hypertensive individuals. J Hum Nutr Diet..

[15] Adams SO, Maller O, Cardello AV (1995). Consumer acceptance of foods lower in sodium. J Am Diet Assoc..

[16] Mattes RD (1997). The taste for salt in humans. Am J Clin Nutr..

[17] Kumanyika SK, Hebert PR, Cutler JA, Lasser VI, Sugars CP, Steffen-Batey L (1993). Feasibility and efficacy of sodium reduction in the Trials of Hypertension Prevention, phase I. Trials of Hypertension Prevention Collaborative Research Group. Hypertension.

[18] Kumanyika SK, Cook NR, Cutler JA, Belden L, Brewer A, Cohen JD (2005). Sodium reduction for hypertension prevention in overweight adults: further results from the Trials of Hypertension Prevention, phase II. J Hum Hypertens..

[19] Bentley B, Lennie TA, Biddle M, Chung ML, Moser DK (2009). Demonstration of psychometric soundness of the Dietary Sodium Restriction Questionnaire in patients with heart failure. Heart Lung.

[20] Sotile WM, Miller HS (1998). Helping older patients to cope with cardiac and pulmonary disease. J Cardiopulm Rehabil.

[21] Rodrigues MP, Rabelo-Silva E, Fuchs FD, Fuchs SC, Moreira LB (2017). Validity and reliability of the dietary sodium restriction questionnaire in patients with hypertension. Eur J Clin Nutr.

[22] Azjen I. Constructing a TPB Questionnaire: conceptual and methodological. 2002. https://people.umass.edu/aizen/pdf/tpb.measurement.pdf. Accessed May 2013.

[23] Welsh D, Marcinek R, Abshire D, Lennie T, Biddle M, Bentley B, Moser D (2010). Theory-based low-sodium diet education for heart failure patients. Home Healthc Nurse.

[24] Chobanian AV, Bakris GL, Black HR, Cushman WC, Green LA, Izzo JL, Joint National Committee on Prevention, Detection, Evaluation, and Treatment of High Blood Pressure (2003). National Heart, Lung, and Blood Institute, National High Blood Pressure Education Program Coordinating Committee, et al. Seventh report of the Joint National Committee on Prevention, Detection, Evaluation, and Treatment of High Blood Pressure. Hypertension.

[25] Sociedade Brasileira de Cardiologia (SBC); Sociedade Brasileira de Hipertensão (SBH); Sociedade Brasileira de Nefrologia (SBN). V Brazilian guidelines for ambulatory monitoring of arterial pressure and III Brazilian guidelines for home monitoring of blood pressure. J Bras Nefrol. 2011;33(3):365–88. 10.1590/s0101-28002011000300013 22042354

[26] Welsh D, Lennie TA, Marcinek R, Biddle MJ, Abshire D, Bentley B, Moser D (2012). Low-sodium diet self-management intervention in heart failure: pilot study results. Eur J Cardiovasc Nurs.

[27] Rodrigues MP. Avaliação da associação de consumo de feijão com arroz e pressão arterial em indivíduos hipertensos em tratamento, Master’s thesis UFRGS—Universidade Federal do Rio Grande do Sul. 2014. https://www.lume.ufrgs.br/handle/10183/127384. Accessed July 2017.

[28] d’Almeida KSM, Souza GC, Rabelo ER (2012). Adaptação Transcultural para o Brasil do Dietary Sodium Restriction Questionnaire (Questionário de Restrição de Sódio na Dieta) (DSRQ). Arq Bras Cardiol.

[29] d’Almeida KSM, Souza GC, Rabelo ER (2013). Validity and reliability of the Dietary Sodium Restriction Questionnaire (DSRQ). Nurs Hosp.

[30] Williams CM, Lovegrove JA, Griffin BA (2012). Dietary patterns and cardiovascular disease. Proc Nutr Soc.

